# Activating autophagy promotes skin regeneration induced by mechanical stretch during tissue expansion

**DOI:** 10.1093/burnst/tkad057

**Published:** 2024-02-07

**Authors:** Jing Du, Wei Liu, Yajuan Song, Yu Zhang, Chen Dong, Shaoheng Xiong, Zhaosong Huang, Tong Wang, Jianke Ding, Qiang He, Zhou Yu, Xianjie Ma

**Affiliations:** Department of Plastic Surgery, Xijing Hospital, Fourth Military Medical University, No. 127 Changle West Road, Xi’an, Shaanxi 710032, China; Key Laboratory of Aerospace Medicine of Ministry of Education, School of Aerospace Medicine, Fourth Military Medical University, No. 169 Changle West Road, Xi’an, Shaanxi 710032, China; Department of Plastic Surgery, Xijing Hospital, Fourth Military Medical University, No. 127 Changle West Road, Xi’an, Shaanxi 710032, China; Department of Plastic Surgery, Xijing Hospital, Fourth Military Medical University, No. 127 Changle West Road, Xi’an, Shaanxi 710032, China; Department of Plastic Surgery, Xijing Hospital, Fourth Military Medical University, No. 127 Changle West Road, Xi’an, Shaanxi 710032, China; Department of Plastic Surgery, Xijing Hospital, Fourth Military Medical University, No. 127 Changle West Road, Xi’an, Shaanxi 710032, China; Department of Plastic Surgery, Xijing Hospital, Fourth Military Medical University, No. 127 Changle West Road, Xi’an, Shaanxi 710032, China; Department of Plastic Surgery, Xijing Hospital, Fourth Military Medical University, No. 127 Changle West Road, Xi’an, Shaanxi 710032, China; Department of Plastic Surgery, Xijing Hospital, Fourth Military Medical University, No. 127 Changle West Road, Xi’an, Shaanxi 710032, China; Department of Plastic Surgery, Xijing Hospital, Fourth Military Medical University, No. 127 Changle West Road, Xi’an, Shaanxi 710032, China; Department of Plastic Surgery, Xijing Hospital, Fourth Military Medical University, No. 127 Changle West Road, Xi’an, Shaanxi 710032, China; Department of Plastic Surgery, Xijing Hospital, Fourth Military Medical University, No. 127 Changle West Road, Xi’an, Shaanxi 710032, China; Department of Plastic Surgery, Xijing Hospital, Fourth Military Medical University, No. 127 Changle West Road, Xi’an, Shaanxi 710032, China

**Keywords:** Autophagy, Mechanical stretch, Skin regeneration, Tissue expansion

## Abstract

**Background:**

Tissue expansion, a technique in which skin regeneration is induced by mechanical stretch stimuli, is commonly used for tissue repair and reconstruction. In this study, we aimed to monitor the autophagy levels of expanded skin after the application of expansion stimuli and explore the effect of autophagy modulation on skin regeneration.

**Methods:**

A rat scalp expansion model was established to provide a stable expanded skin response to mechanical stretch. Autophagy levels at different time points (6, 12, 24, 48 and 72 h after the last expansion) were detected via western blotting. The effect of autophagy regulation on skin regeneration during tissue expansion was evaluated via skin expansion efficiency assessment, western blotting, immunofluorescence staining, TUNEL staining and laser Doppler blood flow imaging.

**Results:**

The autophagic flux reached its highest level 48 h after tissue expansion. Activating autophagy by rapamycin increased the area of expanded skin as well as the thicknesses of epidermis and dermis. Furthermore, activating autophagy accelerated skin regeneration during tissue expansion by enhancing the proliferation of cells and the number of epidermal basal and hair follicle stem cells, reducing apoptosis, improving angiogenesis, and promoting collagen synthesis and growth factor secretion. Conversely, the regenerative effects were reversed when autophagy was blocked.

**Conclusions:**

Autophagy modulation may be a promising therapeutic strategy for improving the efficiency of tissue expansion and preventing the incidence of the complication of skin necrosis.

Highlights

- This study is the first to reveal that mechanical stretch-induced skin regeneration during tissue expansion is accompanied by autophagy induction. We also monitored autophagic flux in the expanded skin at different time points after tissue expansion.
- Activating autophagy accelerated skin regeneration during tissue expansion by enhancing the proliferation and number of epidermal basal and hair follicle stem cells, reducing apoptosis, improving angiogenesis, and promoting collagen synthesis and growth factor secretion.
- Inhibition of autophagy impaired skin regeneration during tissue expansion and even increased the risk of necrosis in the expanded skin.
- Autophagy modulation may be a promising therapeutic strategy for improving the efficiency of tissue expansion and preventing the incidence of the complication of skin necrosis in clinical applications.

## Background

Tissue expansion is one of the most significant surgical procedures for post-burn or trauma repair in plastic surgery. Mechanical stretch mediated by a tissue expander stimulates healthy tissue to regenerate additional skin tissue, which is similar to the adjacent normal skin [1,2]. Although mechanical stretch promotes skin regeneration and enhances epidermal thickness and skin area, insufficient expansion efficiency, its tendency to cause postoperative complications due to the overly thin dermis, and poor blood supply to the expanded skin extremely restrict its practical applications [3,4]. Furthermore, an in-depth understanding of the mechanisms underlying skin regeneration induced by mechanical stretch is lacking [5,6]. Therefore, exploration of the mechanisms underlying skin regeneration induced by mechanical stretch and identification of potential therapeutic targets are necessary.

Autophagy is a cellular process that maintains cell and tissue homeostasis and enhances cell survival during processes such as inflammation, ischemia and stress. Autophagy is involved in various biological processes, such as cell proliferation and apoptosis [7], differentiation [8], angiogenesis [9] and collagen synthesis [10]. It is also involved in tissue regeneration and wound healing [11,12]. Zebrafish caudal fin regeneration is accompanied by changes in autophagy levels [13]. The expression of the autophagy marker microtubule-associated protein 1 light chain 3 (LC3)-II has been found to increase during the initial stage of wound healing in zebrafish models [14]. Moreover, autophagy induction by metformin has been reported to accelerate zebrafish heart regeneration [15]. Therefore, autophagy regulation may serve as a novel strategy for tissue regeneration. Autophagy induction also seems to be essential for hair regeneration, serving as a promising potential hair-loss treatment [16]. In addition, recent studies have suggested that autophagy might be key for explaining how tissues respond to mechanical stretch [17,18]. Autophagy can be induced in osteoblasts and endothelial cells by mechanical stretch stimuli, protecting these cells from damage [19,20]. Mechanical stretch-induced autophagy of periodontal ligament stem cells modulates M1 macrophage polarization and contributes to inflammation-induced bone remodeling and tooth movement processes [21,22]. However, whether autophagy is induced during tissue expansion and its influence on skin regeneration caused by mechanical stretch remain unknown.

We previously designed and established a reliable mouse skin expansion model that provides a stable expanded skin response to mechanical stretch stimuli. We found that the characteristics of the expanded skin in the mouse model were similar to those observed in an expanded human skin [23]. More importantly, many autophagosomes were detected in the expanded skin tissue, suggesting that mechanical stretch had induced autophagy during tissue expansion [24]. These findings laid the foundation for further investigation of the effects of autophagy modulation on skin regeneration during tissue expansion.

To reveal the relationship between autophagy and skin regeneration, we used a rat scalp expansion model and collected the expanded skin at different time points after applying mechanical stretch stimuli. Furthermore, rapamycin (rapa) and/or autophinib [25] were used to regulate autophagy during tissue expansion, and regeneration occurring in the expanded skin was evaluated by assessing cell proliferation and apoptosis, skin stem cell number, angiogenesis, collagen synthesis and growth factor expression. Although tissue expansion has been widely used to perform tissue repair and reconstruction in clinical settings, few targeted therapeutic strategies have been reported to improve regeneration efficiency. Therefore, it is of considerable interest to investigate whether autophagy modulation is a promising therapeutic strategy to facilitate skin regeneration during tissue expansion.

## Methods

### Animals and tissue expansion model

**Figure 1:**
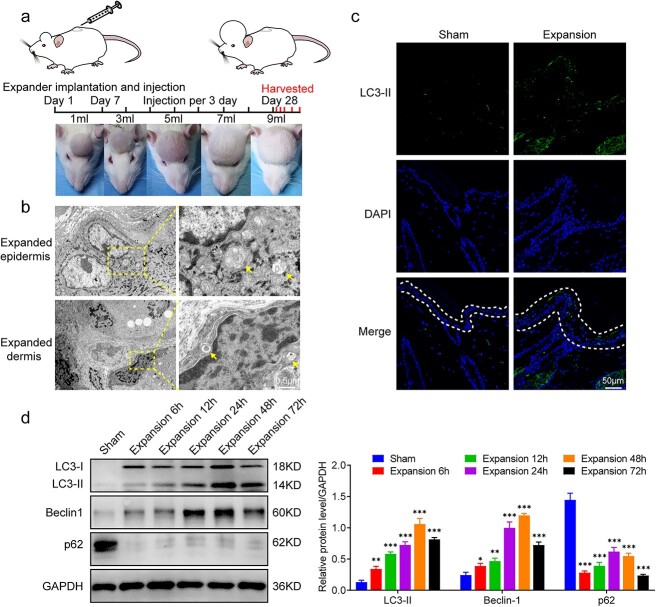
Mechanical stretch induced autophagy during skin tissue expansion. (**a**) Rat scalp tissue expansion model. Expansion was performed every 3 days, and 1 ml of sterile saline was injected into the expander each time. Expanded skins were collected at 6, 12, 24, 48 and 72 h after the last expansion. (**b**) Yellow arrows indicate autophagosomes in an expanded epidermis (upper) and dermis (lower). Left, scale bars: 2 μm; right, scale bars: 0.5 μm. (**c**) Immunofluorescence staining of the autophagy marker LC3-II (green) in the skins obtained from the sham and expansion groups. Scale bars: 50 μm. (**d**) Western blot analysis of the autophagy markers LC3-II, beclin1 and p62 in the expanded skin at different time points after the last expansion. Data are expressed as mean ± SD, n = 3; ^*^p < 0.05, ^*^^*^p < 0.01, ^*^^*^^*^p < 0.001 *vs* the sham group. *LC3 *microtubule-associated protein 1 light chain 3, *DAPI* 4,6-diamino-2-phenyl indole, *Beclin1* beclin 1, autophagy related, *p62* sequestosome 1, *GAPDH* glyceraldehyde-3-phosphate dehydrogenase

Adult Sprague–Dawley (SD) rats (weighing 180–200 g) were purchased from the Experimental Animal Center of the Fourth Military Medical University (Xi’an, China). The procedure for the establishment of a rat scalp expansion model was the same as that used in our previous study [26]. In brief, 36 SD rats were randomly divided into two groups: a sham group (n = 6) and an expansion group (n = 30). A silicone sheet with tissue expanders (Weining, Shanghai, China) was inserted under the scalps of rats in the sham group, but no expansion was performed. In contrast, round 1-ml silicone tissue expanders with a diameter of 1 cm were implanted under the scalps of rats in the expansion group, and the skin was expanded by injecting sterilized saline to flatten the expander (1 ml per injection). The first injection was given immediately after implanting the expanders, and subsequent injections were performed every 3 days from day 7 onward. The expander was enlarged to a volume of 9 ml over 28 days, with the day on which the expander was implanted defined as day 0. All rats were anesthetized, and the expanded skin was collected after the last injection on day 28. Specimens from the sham group were collected 24 h after the last expansion was performed. Specimens from the expansion group were collected 6, 12, 24, 48 and 72 h after the last expansion. At each time point, the expansion group included six rats. All specimens were obtained from the top of the dome of the expanded skin since this region experiences the highest mechanical stretch. The tissue expansion procedure is shown in Figure 1a.

### Rapa and autophinib treatment

To investigate whether and how autophagy influences skin regeneration during tissue expansion, we used rapa (S1039, Selleckchem, USA) and autophinib (S8596, Selleckchem, USA) to modulate autophagic activities in the expanded skin. Autophinib inhibits autophagy, whereas rapa promotes autophagy [25,27]. The animal model was established as described above. An area of 1 × 1 cm^2^ of the scalp was marked on the tissue expander for tracing the expanded skin. Rapa, autophinib or rapa + autophinib were dissolved in DMSO (D8418, Sigma-Aldrich, USA). DMSO (vehicle), rapa, autophinib and rapa + autophinib, were individually mixed with pluronic lecithin organogel [16,28] for topical application on the marked expanded skin at the time of each injection. The rats were divided into the following four groups (n = 6): control group (DMSO treatment), rapa group (1.6 μM), rapa + autophinib group (1.6 μM + 4 mM) and autophinib group (4 mM). The marked expanded skin specimens were harvested 48 h after the last expansion (day 28).

### Histological and morphometric analysis

The harvested skin specimens were fixed in 4% paraformaldehyde for 24 h and embedded in paraffin. They were then cut into 5-μm-thick sections and stained with hematoxylin and eosin (HE staining) and Masson trichrome stain. Histological changes were evaluated by two experienced pathologists. Quantitative analysis of the expanded skin was performed using NDP View2 v. 2.7.25 software (Hamamatsu Photonics K.K, Japan). Five random fields were selected from each scanned image. Epidermal and dermal thicknesses were measured from the HE staining images and collagen volume fraction (CVF) was calculated as the collagen area (Masson stain-positive areas)/total area. All image analyses were conducted using ImageJ software (https://imagej.en.softonic.com/download).

### Skin regeneration efficiency evaluation

The areas and thicknesses of the expanded dermis and epidermis of the marked expanded HE-stained skin were measured. CVF was also calculated. These measures were used to evaluate skin regeneration efficiency.

### Transmission electron microscopy

Samples for transmission electron microscopy (TEM) were prepared as previously described [24]. The skins collected from the sham and expansion groups were cut into 1-mm^3^ pieces and placed in a precooled 2.5% buffered glutaraldehyde solution for 24 h at 4°C. The tissue was then washed, fixed, stained with uranyl acetate, dehydrated using alcohol and propylene oxide and embedded in epoxy resin. Next, 800-nm-thick sections were cut and stained with toluidine blue. The fields of the expanded epidermis and dermis were selected as the observation area. Lastly, the specimens were cut into 90-nm ultrathin sections, stained with uranyl acetate and lead citrate, and observed using a JEM-1400 transmission electron microscope (JEOL, Tokyo, Japan).

### Western blotting

Protein expression levels in the expanded skin were determined using western blotting. Tissue samples were lysed in 300 μl of radioimmunoprecipitation assay buffer (CoWin Biosciences, Taizhou, China) to extract proteins. Total protein concentrations were determined using a BCA protein assay kit (CoWin Biosciences, Taizhou, China). For each specimen, 15 μg of protein was separated using 7.5, 12.5 or 15% SDS-PAGE (Bio-Rad Laboratories, Hercules, CA) and then transferred onto polyvinylidene difluoride membranes (Millipore, Billerica, MA, USA). The membranes were blocked using PBS with 0.1% Tween 20 containing 5% milk powder at room temperature for 1 h and then incubated with different antibodies. The primary antibodies used were as follows: rabbit anti- Glyceraldehyde-3-phosphate dehydrogenase (10494–1-AP, Proteintech, China) (1 : 5000), rabbit anti-LC3-II (18725–1-AP, Proteintech, China) (1 : 300), rabbit anti-beclin 1, autophagy related (beclin1; 11306–1-AP, Proteintech, China) (1 : 3000), rabbit anti-sequestosome 1 (p62; 18420–1-AP, Proteintech, China) (1 : 3000), rabbit anti- B-cell lymphoma 2 (BCL2)-associated X protein (BAX; 50 599–2-Ig, Proteintech, China) (1 : 5000), rabbit anti-BCL-2 (12 789–1-AP, Proteintech, China) (1 : 3000), rabbit anti-caspase 3, apoptosis-related cysteine peptidase (caspase 3; 19677–1-AP, Proteintech, China) (1 : 1000), rabbit anti-collagen, type I (col I; 14695–1-AP, Proteintech, China) (1 : 2000), rabbit anti-col III (22734–1-AP, Proteintech, China) (1 : 1000), basic fibroblast growth factor (bFGF; ab208687, Abcam, UK) (1 : 1000), rabbit anti-connective tissue growth factor (CTFG; 23 936–1-AP, Proteintech, China) (1 : 1000), rabbit anti-transforming growth factor (TGF-β; 21 898–1-AP, Proteintech, China) (1 : 1000), rabbit anti-epidermal growth factor (EGF; ab184265, Abcam, UK) (1 : 1000) and rabbit anti-vascular endothelial growth factor (VEGF; ab32152, Abcam, UK) (1 : 3000). Subsequently, the membranes were incubated with the secondary antibodies CW0103 and CW0102 (CoWin Biosciences, China) (1:3000). Blots were detected using an ECL kit (Thermo Fisher Scientific, Waltham, MA, USA) and quantified using the ImageJ software.

### TUNEL staining

For quantifying apoptosis occurring in the expanded skin from each group, sections were treated according to the manufacturer’s instructions and TUNEL staining was performed using a DAB (SA-HRP) TUNEL cell apoptosis detection kit (G1507, Servicebio, China) according to the manufacturer’s manual. In brief, the expanded skin tissue sections were permeabilized with proteinase K and then incubated with the TUNEL mixture for 1 h at 37°C. Finally, the DAB chromogenic reagent (G1212, Servicebio, China) was used to stain TUNEL-positive cells. We randomly selected six high-magnification images from each specimen. The apoptosis rate within the expanded skin was calculated by counting TUNEL-positive cells and total cells per field.

### Laser Doppler blood flow imaging

To evaluate the blood supply and vascular flow in the expanded skin via laser Doppler blood flow imaging, we scanned the entire expanded scalp skin tissue of each expansion model rat on day 28. Perfusion units (PUs) were considered indicative of microcirculatory blood flow. The PUs of the marked expanded area were assayed using MoorFLPI software v. 3.0 (Moor Instruments, Devon, UK). We evaluated each animal 10 times and calculated the mean value.

### Immunofluorescence staining

After dewaxing, hydration and antigen retrieval, the tissue sections were blocked with PBS containing 5% BSA for 1 h. The primary antibodies rabbit anti-LC3II (18725–1-AP, Proteintech, China) (1 : 200), rabbit anti-cytokeratin 14 (CK14; 14, 10 143–1-AP, Proteintech, China) (1 : 400), rabbit anti-CK10 (ab76318, Abcam, UK) (1 : 150), rabbit anti-CK15 (ab52816, Abcam, UK) (1:100), mouse anti-proliferating cell nuclear antigen (PCNA; ab29, Abcam, UK) (1 : 500) and rabbit anti-CD34 (ab81289, Abcam, UK) (1 : 200) were incubated overnight at 4°C in a humid chamber. Fluorescent dye-conjugated secondary antibodies, namely, Andy fluor 488 goat anti-rabbit IgG (H + L) antibody (L110A, ABP Biosciences, USA) (1 : 1000), Andy fluor 594 goat anti-mouse IgG (H + L) antibody (L119A, ABP Biosciences, USA) (1 : 1000) and Andy fluor 594 goat anti-rabbit IgG (H + L) antibody (L120A, ABP Biosciences, USA) (1 : 1000), were incubated for 1 h at room temperature. The cell nuclei were counterstained with 10 μg/ml of 4,6-diamino-2-phenyl indole (DAPI; Invitrogen, Carlsbad, CA, USA). Finally, images were captured using a confocal fluorescence microscope (Nikon, Tokyo, Japan). To assess angiogenesis in the expanded skin, vascular density was quantified as the average number of CD34^+^ vessels in six random fields.

### Statistical analysis

Statistical analyses were performed using GraphPad Prism v. 8.3.1 software (GraphPad, USA). Measurement data are presented as mean ± standard deviation. Comparisons between two groups were conducted using Student’s *t*-test, whereas comparisons among multiple groups were assessed using one-way analysis of variance. Tukey’s test was used for *post hoc* multiple comparisons after one-way analysis of variance. A *p*-value < 0.05 was considered statistically significant.

## Results

### Mechanical stretch induces autophagy during tissue expansion

To investigate whether autophagy is induced by mechanical stretch during tissue expansion, we created a rat model of scalp tissue expansion (Figure 1a). We harvested expanded and nonexpanded skin and observed typical autophagosomes in both keratinocytes from the epidermis and fibroblasts from the dermis using TEM (Figure 1b). Autophagosomes were easier to observe in expanded skin than non-expanded skin and autophagosome formation is considered a marker of autophagy [29]. In addition, immunofluorescence staining showed greater LC3-II expression in expanded skin than in nonexpanded skin. A large amount of punctate fluorescence was observed in both the expanded epidermis and dermis (Figure 1c), indicating that autophagy had been induced by mechanical stretch during tissue expansion. We also monitored dynamic autophagic flux in the expanded skin at different time points after tissue expansion using western blot analysis of LC3-II, beclin1 and p62. Under mechanical stretch stimulation, the expression of LC3-II (*p* < 0.01) and beclin1 (*p* < 0.05) significantly increased, whereas that of p62 decreased (*p* < 0.001, Figure 1d). The autophagic flux reached its highest level 48 h after tissue expansion (Figure 1d). These results demonstrate that mechanical stretch during tissue expansion can induce autophagy in a time-dependent manner.

### Activating autophagy promotes skin regeneration during tissue expansion

**Figure 2:**
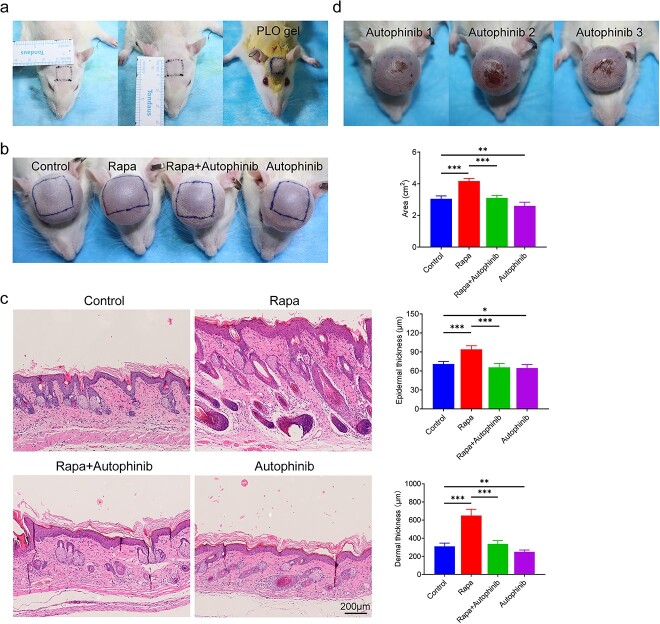
Rapamycin promotes skin regeneration during tissue expansion, whereas autophinib inhibits skin regeneration. (**a)** An area of 1 × 1 cm^2^ was marked on rat scalps. The marked area of the expanded skin was treated with rapamycin (1.6 μM), rapa + autophinib (1.6 μM + 4 mM) or autophinib (4 mM), each dissolved in DMSO and added to PLO gel or vehicle control (DMSO in 100 μl of PLO gel) when each expansion was performed. (**b**) Representative expanded skin areas from the control, rapa, rapa + autophinib and autophinib groups after 28 days of expansion. The marked area was measured for each group (n = 6). (**c**) Hematoxylin and oesin staining of the expanded skin from the control, rapa, rapa + autophinib and autophinib groups showed that activating autophagy thickens the expanded epidermis and dermis, whereas inhibiting autophagy causes thinning of the expanded dermis. Scale bars: 200 μm. **d** Half of the expanded flaps developed necrosis and scabs in the expanded skin in the autophinib group. This was not observed in any of the other groups. Data are expressed as the mean ± SD, n = 6; ^*^p < 0.05, ^*^^*^p < 0.01, ^*^^*^^*^p < 0.001. *PLO* pluronic lecithin organogel, *Rapa* rapamycin

**Figure 3:**
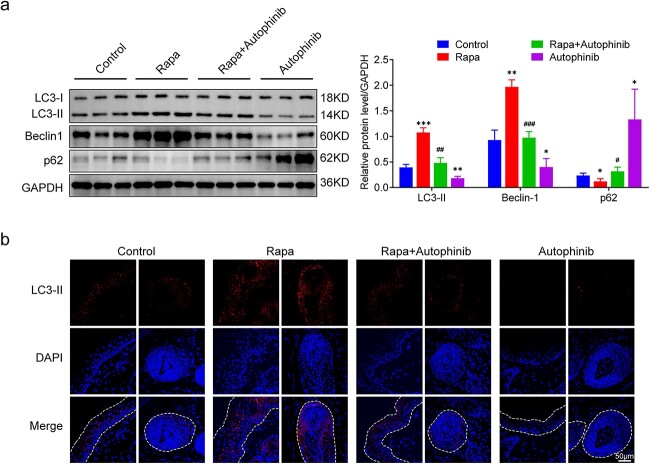
Detection of autophagic flux in the expanded skin. (**a**) Western blot analysis of the autophagy markers LC3-II, beclin1 and p62 in the expanded skin obtained from the control, rapa, rapa + autophinib and autophinib groups, n = 3. (**b**) Immunofluorescence staining of the autophagy marker LC3-II (red) in the expanded skin. The expanded epidermis and hair follicle cells where LC3-II was mainly expressed are marked by dash lines. Scale bars: 50 μm. Data are expressed as mean ± SD. ^*^p < 0.05, ^*^^*^p < 0.01, ^*^^*^^*^p < 0.001 *vs* the control group. ^#^*p* < 0.05, ^##^*p*<0.01, ^###^*p* < 0.001 *vs* the rapa group. *LC3* microtubule-associated protein 1 light chain 3, *DAPI* 4,6-diamino-2-phenyl indole, *Beclin1* beclin 1, autophagy related, *p62* sequestosome 1, *GAPDH* glyceraldehyde-3-phosphate dehydrogenase, *Rapa* rapamycin

To explore the effects of autophagy on skin regeneration during tissue expansion, the expanded rat scalps were treated with DMSO (control), rapa, rapa and autophinib (rapa + autophinib) or autophinib. The areas and thicknesses of the marked expanded skins were measured to evaluate skin regeneration efficiency (Figure 2a). In the rapa group (4.17 ± 0.15 cm^2^), the average area was larger than that in the DMSO group (3.04 ± 0.17 cm^2^, *p* < 0.001) and the effect was reversed by autophinib (3.10 ± 0.14 cm^2^, *p* < 0.001, Figure 2c). Similarly, both the epidermis (94.17 ± 5.08 μm) and dermis (649.33 ± 63.74 μm) of the rapa group were thicker than those of the DMSO group (71.00 ± 3.56 μm and 310.00 ± 32.54 μm, respectively, *p* < 0.001, Figure 2d). Compared with other groups, the autophinib group showed smallest areas and thinnest epidermis and dermis. These results demonstrated that autophagy activation can promote skin regeneration during tissue expansion, whereas autophinib inhibits skin regeneration. Moreover, in the autophinib group, half of the expanded skins developed flap necrosis and scabs (Figure 2b). As shown in Figure 3a, the expression of LC3-II (*p* < 0.001) and beclin1 (*p* < 0.01) was upregulated and that of p62 (*p* < 0.05) was downregulated in the rapa group. Conversely, autophinib treatment lowered LC3-II (*p* < 0.05) and beclin1 (*p* < 0.05) expression and elevated p62 expression (*p* < 0.05). Immunofluorescence staining of LC3-II in the expanded skin showed similar results (Figure 3b). It should be noted that LC3-II-positive puncta were primarily localized in the epidermis and hair follicles, where cells proliferated vigorously. These findings suggest that skin regeneration during tissue expansion is closely associated with autophagy activity. Activating autophagy accelerated skin regeneration during tissue expansion, whereas autophagy inhibition suppressed it.

### Activating autophagy facilitates cell proliferation and impedes apoptosis

**Figure 4:**
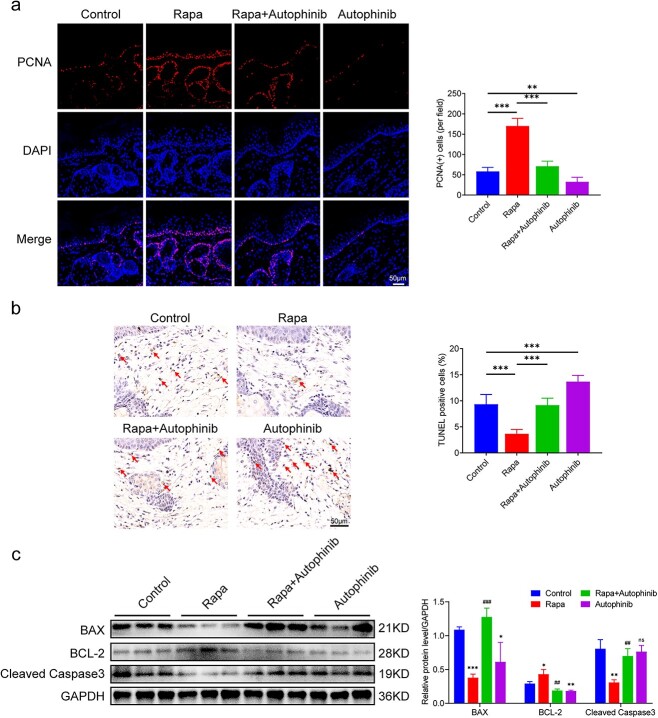
Activating autophagy facilitates cell proliferation and reduces apoptosis in the expanded skin. (**a**) Immunofluorescence staining of PCNA and quantification of PCNA^+^ cells (red) in the expanded skin obtained from the control, rapa, rapa + autophinib and autophinib groups. Scale bars: 50 μm. (**b**) TUNEL staining and rates of TUNEL-positive cells (red arrows) in each group. Scale bars: 50 μm. Cell counts from six randomly selected visual fields in each group, n = 6; ^*^^*^p < 0.01, ^*^^*^^*^p < 0.001. (**c**) Western blot analysis of the apoptosis-associated proteins BAX, BCL-2 and cleaved caspase 3 in the expanded skin obtained from each group, n = 3. Data are expressed as mean ± SD. ^*^p < 0.05, ^*^^*^p < 0.01, ^*^^*^^*^p < 0.001 *vs* the control group. ^##^*p* < 0.01, ^###^*p* < 0.001* vs* the rapa group. ns, Not significant. *PCNA* proliferating cell nuclear antigen, *DAPI* 4,6-diamino-2-phenyl indole, *Rapa* rapamycin, *BAX* BCL2-associated X protein, *BCL-2* B-cell CLL/lymphoma 2, *GADPH* glyceraldehyde-3-phosphate dehydrogenase

To evaluate the effects of autophagy modulation during tissue expansion on cell proliferation and apoptosis, immunohistochemical staining of PCNA in the expanded skin was performed. As shown in Figure 4a, PCNA^+^ cells were distributed throughout all layers of the expanded skin, but predominantly in the basal layer of the epidermis and hair follicles. We observed significantly more PCNA^+^ cells per field in the rapa group (170 ± 17) than in the control group (58 ± 9, *p* < 0.001, Figure 4a). The lowest number of PNCA^+^ cells was observed in the autophinib group (33 ± 10, *p* < 0.01). Interestingly, the rapa group contained more than one layer of PCNA^+^ cells in the basal layer of the epidermis. We then used TUNEL staining to determine the proportions of apoptotic cells. The rapa-treated expanded skin (3.67% ± 0.75%) had the fewest apoptotic cells (*p* < 0.001), whereas the autophinib-treated expanded skin (13.67 ± 1.11%) had the highest number of apoptotic cells (*p* < 0.001, Figure 4b). We performed western blot analysis to measure the expression of apoptosis-related proteins, including BCL-2, BAX and cleaved caspase 3, in the expanded skin. The expression of BAX (*p* < 0.001) and cleaved caspase 3 (*p* < 0.01) in the rapa group was lower than that in the control group, whereas the expression of BCL-2 (*p* < 0.05) in the rapa group was higher than that in the control group (Figure 4c). Overall, these data suggest that autophagy activation facilitates cell proliferation and reduces apoptosis, whereas autophagy inhibition has the opposite effect.

### Activating autophagy increases number of stem cells during tissue expansion

**Figure 5:**
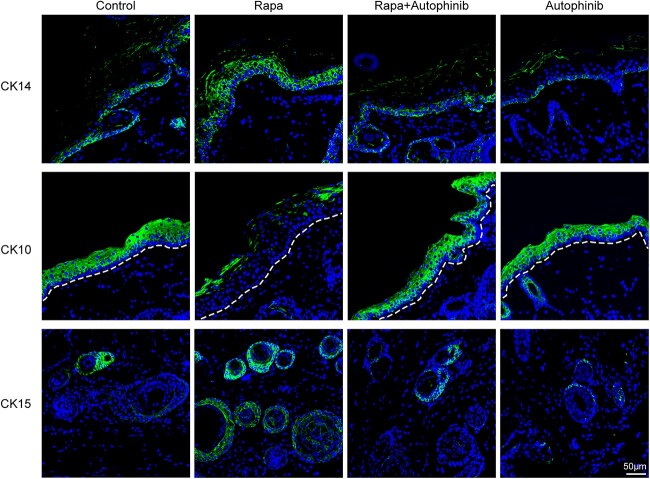
Activating autophagy enhances the number of epidermal basal stem cells and hair follicle stem cells in the expanded skin. Both CK14 (green) (a marker for epidermal basal stem cells) and CK15 (green) (a marker for hair follicle stem cells) expression in the expanded skin obtained from the rapa group was higher than in the expanded skin obtained from other groups, with the autophinib group showing the lowest expression. CK10 (green) (which is found in differentiated keratinocytes) expression in the expanded skin obtained from the rapa group decreased in the expanded epidermis. Scale bars: 50 μm, n = 6. *Rapa* rapamycin, *CK14* cytokeratin 14

Next, we detected the number of epidermal basal stem cells and hair follicle stem cells, considering the close association of stem cells with tissue regeneration. CK14 and CK15 are markers of epidermal basal stem cells and hair follicle stem cells [30,31], respectively. CK10 is a marker of keratinocytes in the suprabasal layers of mature epidermal cells [32]. Immunofluorescence staining confirmed that CK14 expression in the epidermis and CK15 expression in the dermis of the rapa group skin were higher than those in the control and the rapa + autophinib groups, as shown in Figure 5. Among the four groups, the autophinib group showed the lowest expression of CK14 and CK15. Meanwhile, CK10 expression in the expanded epidermis of the rapa group was extremely low compared with that in the expanded epidermis of the other groups (Figure 5). These results suggest that autophagy activation increases the number of epidermal basal stem cells and hair follicle stem cells in the expanded skin during tissue expansion, whereas autophagy inhibition reduces the number of stem cells.

### Activating autophagy promotes angiogenesis in the expanded skin

**Figure 6:**
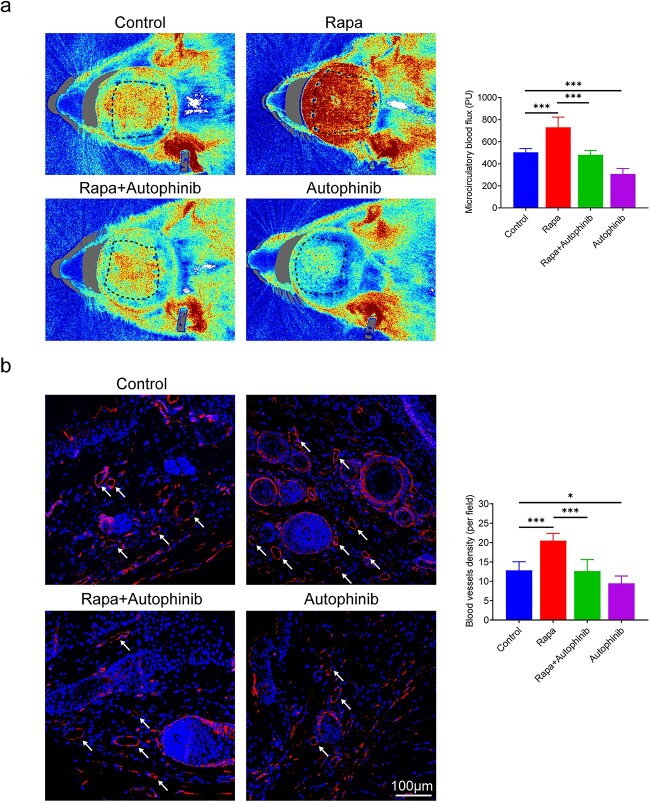
Activating autophagy promotes angiogenesis in the expanded skin. (**a**) Laser Doppler perfusion images of the marked areas of the expanded skin obtained from each group and the quantification of microcirculatory blood flow, n = 6. (**b**) Immunofluorescence staining of CD34^+^ blood vessels (white arrows) and blood vessel density counts. Scale bars: 100 μm, n = 6. Data are expressed as mean ± SD. ^*^p < 0.05, ^*^^*^^*^p < 0.001. *Rapa* rapamycin, *PU* perfusion unit

Angiogenesis is closely related to tissue regeneration. To assess blood perfusion of expanded skin during autophagy activation or suppression, laser Doppler blood flow imaging was implemented. The marked areas of the expanded skin from the rapa group (730 ± 86 PU) showed greater microcirculatory blood perfusion than those from the control (504 ± 30 PU, *p* < 0.001) and rapa + autophinib (481 ± 36 PU, *p* < 0.001, Figure 6a) groups, whereas the autophinib group showed the lowest blood perfusion (307 ± 46 PU, *p* < 0.001, Figure 6a). Immunohistochemical staining of CD34 was used to calculate the blood vessel densities of the expanded skin. The highest number of CD34^+^ blood vessels (white arrows) was observed in the rapa group (21 ± 2, *p* < 0.001) and the lowest number was observed in the autophinib group (10 ± 2, *p* < 0.05, Figure 6b). These findings demonstrate that autophagy activation promotes angiogenesis and autophagy inhibition impairs angiogenesis during tissue expansion.

### Activating autophagy enhances collagen synthesis in the expanded skin

**Figure 7:**
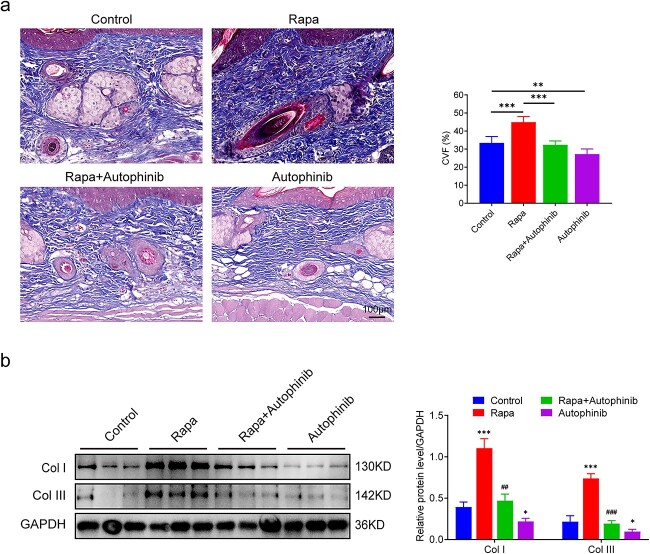
Activating autophagy enhances collagen synthesis in the expanded skin. (**a**) Masson trichrome staining of the expanded skin and a comparison of the CVF of each group, n = 6, ^**^p < 0.05, ^***^p<0.001. Scale bar: 100 µm (**b**) Western blot analysis of type I and type III collagen in the expanded skin obtained from each group, n = 3. Data are expressed as mean ± SD. ^*^p< 0.05, ^*^^*^p < 0.01, ^*^^*^^*^p < 0.001 *vs* the control group. ^##^*p* < 0.01, ^###^*p*<0.001 *vs* the rapa group. *Rapa* rapamycin, *CVF* collagen volume fraction, *Col I* collagen type I, *GADPH* glyceraldehyde-3-phosphate dehydrogenase

Collagen synthesis determines the thickness of the dermis in the expanded skin. Therefore, we used Masson trichrome staining to measure collagen content and CVF. CVF was highest in the rapa group (44.9 ± 2.84%, *p* < 0.001) and lowest in the autophinib group (27.26 ± 2.59%, *p* < 0.01, Figure 7a). Western blotting was used to detect the expression of col I and col III. The expression of both collagens in the expanded skin of the rapa group was significantly higher than that in the expanded skin of the control group (*p* < 0.001). The lowest expression of both collagens was observed in the autophinib group (*p* < 0.05, Figure 7b). Thus, autophagy activation facilitates collagen synthesis during tissue expansion and autophagy inhibition reduces collagen synthesis.

### Activating autophagy increases the expression of growth factors in the expanded skin

**Figure 8:**
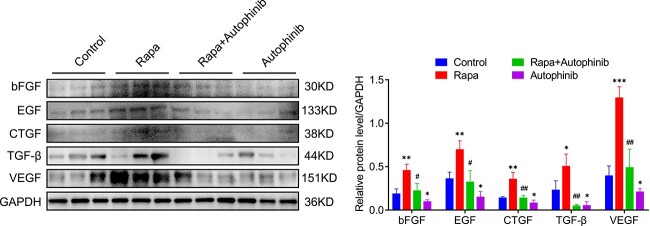
Activating autophagy increases the expression of growth factors in the expanded skin. Western blot analysis of bFGF, EGF, CTGF, TGF-β and VEGF in the expanded skin obtained from each group. The protein expression of the growth factors was greater in the rapa group than in the control and rapa + autophinib groups. The autophinib group showed lower expression of bFGF, EGF, CTGF, TGF-β and VEGF in the expanded skin than the control group. n = 3. Data are expressed as mean ± SD. ^*^p < 0.05, ^*^^*^p < 0.01, ^*^^*^^*^p < 0.001 *vs* the control group. ^#^*p*<0.05, ^##^*p* < 0.01 *vs* the rapa group. *Rapa* rapamycin, *EGF* epidermal growth factor, *VEGF* vascular endothelial growth factor, *bFGF* basic fibroblast growth factor, *CTGF* connective tissue growth factor, *TGF-β* transforming growth factor β, *GADPH* glyceraldehyde-3-phosphate dehydrogenase

The expression of bFGF, EGF, CTGF, TGF-β and VEGF in skin tissues is strongly correlated to skin regeneration during tissue expansion [2,33]. Hence, we performed western blotting to examine the expression of these proteins. As shown in Figure 8, the expression of bFGF, EGF, CTGF, TGF-β and VEGF was significantly elevated in rapa-treated expanded skin compared with DMSO- and rapa + autophinib-treated expanded skin (*p* < 0.05). Moreover, reduced expression of these proteins was observed in autophinib-treated expanded skin (*p* < 0.05, Figure 8). Thus, autophagy activation enhances the levels of the growth factors bFGF, EGF, CTGF, TGF-β and VEGF in the expanded skin.

## Discussion

Harvesting the regenerative expanded skin after tissue expansion is an important therapeutic strategy for the repair and reconstruction of tissue defects in plastic surgery. However, insufficient tissue expansion efficiency and poor expanded skin quality owing to low blood supply and an overly thin expanded dermis make it a challenging process [3,4]. It is essential to elucidate the mechanism by which skin regeneration is induced by mechanical stretch and identify methods for promoting the efficiency of expansion and the quality of the expanded skin. Recent studies have shown that autophagy is closely related to tissue regeneration and wound healing [11,12] and is a potential mediator of the mechanical forces involved in tissue expansion [17,18,22]. Previously, we created a stable mouse model of tissue expansion in which we observed the presence of autophagosomes in the expanded skin [23,24]. In the present study, we focused on determining the role of autophagy in skin regeneration induced by mechanical stretch during tissue expansion and the effects of autophagy modulation on tissue expansion efficiency and expanded skin quality.

Autophagy was found to mediate skin regeneration during tissue expansion. In this study, TEM revealed the presence of autophagosomes in both the expanded epidermis and dermis, and the increased expression of LC3-II in the expanded skin, especially in the keratinocytes and hair follicle cells, which are capable of active proliferation. Autophagy was induced during tissue expansion, indicating that the mechanical stretch exerted by tissue expansion induces autophagy. By monitoring the autophagic flux through the detection of LC-II expression, it is possible to evaluate the level of autophagic activation [29]. By conducting further investigation, we found that autophagic activity shows a time-dependent pattern after receiving mechanical stretch stimuli. These data suggest that mechanical stretch can induce autophagy, and autophagy may be a key mediator of mechanical stretch and skin regeneration during tissue expansion.

Autophagy is closely associated with tissue regeneration [12,34]. A recent study found that metformin-induced autophagy accelerates zebrafish heart regeneration [15]. In addition, autophagy has been shown to be necessary for caudal fin regeneration, and the initial stages of caudal fin regrowth coincide with autophagy activation [11,13]. To investigate the role of mechanical stretch-induced autophagy in tissue expansion and the influence of autophagy regulation on expansion efficiency, we selected the autophagy modulators rapa and autophinib to activate and inhibit autophagic activity during tissue expansion, respectively. Our results showed that activating autophagy enlarges the area of the expanded skin and increases the thickness of both the epidermis and dermis. Conversely, blocking autophagy diminishes the area of the expanded skin and the thickness of the dermis and even leads to necrosis and scab formation in the expanded skin. These findings indicate that autophagy is necessary for skin regeneration during tissue expansion. Activating autophagy beyond the levels at which it occurs naturally can further promote skin regeneration efficiency. Recent studies have suggested that modulating autophagy can contribute to tissue regeneration. Age-dependent autophagy induction can promote axon regeneration by inhibiting the NOTCH pathways [35]. Re-establishing autophagy was found to reverse senescence and restore regenerative functions in geriatric satellite cells and foster muscle regeneration in sarcopenia [36]. Research has found that the activation of mTOR-independent autophagy shows promise for promoting liver regeneration [37]. Therefore, autophagy modulation may serve as a potential novel therapeutic strategy for the promotion of skin regeneration during tissue expansion.

It is well known that stem cells in the basal layers of the epidermis and hair follicle stem cells contribute to skin regeneration [38]. In cutaneous cells, a homeostatic relationship between cell proliferation and apoptosis is observed [39]. Studies have confirmed that autophagy plays a key role in maintaining the dynamic balance among cell proliferation, differentiation and apoptosis [7,40,41]. We revealed that the activation of autophagy using rapa promotes the proliferation of both epidermal basal cells and dermal hair follicle cells in the expanded skin. Most strikingly, 2–3 layers of PCNA^+^ cells were observed in the epidermal basal layer of the expanded skin in the rapa group. Therefore, enhanced autophagy levels likely contribute to skin regeneration during expansion by promoting epidermal cell proliferation and migration [42]. Furthermore, apoptosis detected via TUNEL staining and apoptosis-related proteins identified via western blot analysis showed that activating autophagy reduces apoptosis in the expanded skin. Autophagy may regulate apoptosis by affecting endocytosis [43]. Inhibiting autophagy hinders the effects of mechanical stretch and/or rapa-induced autophagy on cell proliferation and apoptosis, suggesting that autophagy is necessary for skin regeneration induced by mechanical stretch during tissue expansion. CK14 is a type II keratin and a stem cell marker in the basal layer of the epidermis [31], whereas CK10 occurs in abundance in the terminally differentiated keratinocytes of the epidermis and can inhibit the proliferation of human keratinocytes [44]. Rapa-treated expanded skin showed higher CK14 expression and lower CK10 protein levels in the expanded epidermis. This indicates that the number of epidermal stem cells was increased in the epidermis via the activation of autophagic activity during tissue expansion. Conversely, blocking autophagy in the expanded skin treated with autophinib curbed the number of epidermal basal stem cells. CK15 is a marker of hair follicle stem cells [45]; CK15 was expressed most abundantly in the rapa group and least abundantly in the autophinib group. The number of epidermal basal stem cells and dermal hair follicle stem cells increased when autophagic activity was enhanced during tissue expansion. More importantly, the number of stem cells is directly related to tissue regeneration potential [46]. These results indicate that mechanical stretch-induced autophagy is necessary for expanded skin regeneration, and activating autophagy further promotes skin regeneration during tissue expansion by facilitating cell proliferation, reducing apoptosis, and increasing the number of epidermal basal stem cells and dermal hair follicle stem cells in the expanded skin.

In addition to insufficient expansion efficiency, a high incidence of complications during tissue expansion is a barrier to clinical application [47,48]. Necrosis of the expanded skin is catastrophic for patients undergoing a prolonged period of tissue expansion therapy. A poor blood supply to the regenerated skin and an overly thin expanded dermis are likely to be responsible for ulcers and necrosis of the expanded skin [49]. We investigated the effects of autophagy modulation on angiogenesis and collagen synthesis during tissue expansion. Blood vessels are essential components of almost all tissues and organs [50] and provide oxygen and nutrients for various cellular functions, including tissue regeneration [51]. Accordingly, better vascularization enhances skin wound healing and tissue regeneration. We found that autophagy activation by rapa during tissue expansion increased vascular perfusion and blood vessel density in the expanded skin. An improved blood supply not only directly contributes to skin regeneration but also reduces the risk of skin necrosis. Col I and col III proteins are the first and second most abundant structural proteins in the skin [52]. Col III is a crucial component of the extracellular matrices that link mechanical stimuli to tissue regeneration [53]. Our findings from Masson staining showed that activating autophagy accelerated collagen deposition during skin regeneration, whereas blocking autophagy reversed this effect. In addition, col I and col III were expressed the most in the rapa group and the least in the autophinib group. This indicates that autophagy induction is necessary for collagen synthesis during tissue expansion. More importantly, necrosis occurred in half of the expanded skins of the autophinib group in our study. This can be attributed to the lower levels of collagen deposition and the poor blood supply resulting from autophagy inhibition during tissue expansion. Thus, promoting collagen synthesis and angiogenesis of expanded skin by activating autophagy is of clinical significance.

Various growth factors have been found to participate in cell proliferation, angiogenesis, and collagen synthesis and deposition during tissue expansion [33,54]. Furthermore, autophagic activity is closely related to the expression of these growth factors [55]. In the present study, increased expression of bFGF, EGF, CTGF, TGF-β and VEGF was observed in the expanded skin treated with rapa. EGF secreted by skin stem cells can regulate stem cell functions and promote cell regeneration [56]. Elevating EGF levels by activating autophagy may accelerate skin regeneration by promoting cell proliferation in the expanded skin and enhancing the number of the epidermal basal and hair follicle stem cells. VEGF expression can be stimulated by bFGF, and they then work together to mediate tissue angiogenesis [57]. Promoting the expression of bFGF also accelerates wound healing [58]. In our study, autophagy activation increased the expression of VEGF and bFGF. This may have contributed to an improved blood supply to the expanded skin during tissue expansion and thereby accelerated skin regeneration. CTGF is a profibrotic factor that plays a vital role in the induction of collagen deposition by mediating TGF-β activity [59]. TGF-β is one of the main regulators of collagen synthesis and deposition [60]. Our study showed that activating autophagy increases CTGF and TGF-β secretion, thereby promoting collagen synthesis and deposition in the expanded skin during tissue expansion. Other than the direct effects of autophagy modulation on growth factors, the increased number of epidermal basal and hair follicle stem cells contributes to the secretion of growth factors. Therefore, activating autophagy may accelerate skin regeneration during tissue expansion through upregulation of growth factor expression.

## Conclusions

Our study demonstrated that mechanical stretch-induced autophagy is highly involved in skin regeneration during tissue expansion. Autophagy inhibition impairs skin regeneration by reducing the proliferation of epidermal basal and hair follicle stem cells, decreasing angiogenesis and collagen synthesis in the expanded skin and reducing the secretion of growth factors. Moreover, the resulting poor blood supply and thinning of the expanded dermis can lead to necrosis. In clinical practice, severe skin-related complications, especially necrosis of the expanded flap, lead to treatment interruption, which is undoubtedly catastrophic for patients. Therefore, autophagy modulation is crucial for the reduction of skin necrosis during tissue expansion. Autophagy activation promotes skin regeneration during tissue expansion by enhancing the proliferation and number of epidermal basal and hair follicle stem cells, reducing apoptosis and promoting angiogenesis, collagen synthesis and growth factor secretion in the expanded skin. Thicker expanded skins with a larger area are expected to address issues of insufficient expansion efficiency. These benefits suggest that autophagy modulation is a suitable therapeutic strategy for improving the efficiency of tissue expansion and has considerable clinical value for preventing the incidence of the complication of skin necrosis.

## Abbreviations

BAX: BCL2-associated X protein; BCL-2: B-cell CLL/lymphoma 2; Beclin-1: Beclin 1, autophagy related; bFGF: Basic fibroblast growth factor; caspase 3: Caspase 3, apoptosis-related cysteine peptidase; CK14: Cytokeratin 14; col I: Collagen, type I; CTGF: Connective tissue growth factor; CVF: Collagen volume fraction; DAPI: 4,6-Diamino-2-phenyl indole; DMSO: Dimethyl sulfoxide; EGF: Epidermal growth factor; GAPDH: Glyceraldehyde-3-phosphate dehydrogenase; HE: Hematoxylin and eosin; LC3: Microtubule-associated protein 1 light chain 3; PCNA: Proliferating cell nuclear antigen; PLO: Pluronic lecithin organogel; PU: Perfusion units; p62: Sequestosome 1; Rapa: Rapamycin; SD: Sprague–Dawley; TEM: Transmission electron microscopy; TGF-β: Transforming growth factor β; VEGF: Vascular endothelial growth factor.

## Funding

This work was supported by grants from the National Natural Science Foundation of China (82172229, 81971851, and 82102347), the Natural Science Basic Research Plan in Shaanxi Province of China (2022JM-600) and the Foundation of Xijing Hospital of Fourth Military Medical University Grants (XJZT21CM33).

## Authors’ contributions

JD and WL contributed equally to this work. JD, ZY and XM designed and conceived the experiments. JD, WL, YS, CD, YZ, SX, ZH and JDing performed all experiments. JD, WL, CD, TW and QH analyzed the data. JDing, ZY and XM wrote and edited the manuscript. ZY and XM equally supervised the study. All authors commented on and approved the final manuscript.

## Ethics approval and consent to participate

All animal experiments were approved by the Experimental Animal Committee of the Fourth Military Medical University (permit no. IACUC-20210206) and performed in accordance with the guidelines of the National Institutes of Health Guide for the Care and Use on laboratory animals.

## Conflict of interest

None declared.

## Data availability

All data and materials supporting the findings of this study are available from the corresponding authors upon reasonable request.
